# Does trust in health care influence the use of complementary and alternative medicine by chronically ill people?

**DOI:** 10.1186/1471-2458-6-188

**Published:** 2006-07-18

**Authors:** A van den Brink-Muinen, PM Rijken

**Affiliations:** 1 NIVEL (Netherlands institute for health services research), Utrecht, The Netherlands

## Abstract

**Background:**

People's trust in health care and health care professionals is essential for the effectiveness of health care, especially for chronically ill people, since chronic diseases are by definition (partly) incurable. Therefore, it may be understandable that chronically ill people turn to complementary and alternative medicine (CAM), often in addition to regular care. Chronically ill people use CAM two to five times more often than non-chronically ill people. The trust of chronically ill people in health care and health care professionals and the relationship of this with CAM use have not been reported until now. In this study, we examine the influence of chronically ill people's trust in health care and health care professionals on CAM use.

**Methods:**

The present sample comprises respondents of the 'Panel of Patients with Chronic Diseases' (PPCD). Patients (≥25 years) were selected by GPs. A total of 1,625 chronically ill people were included. Trust and CAM use was measured by a written questionnaire. Statistical analyses were t tests for independent samples, Chi-square and one-way analysis of variance, and logistic regression analysis.

**Results:**

Chronically ill people have a relatively low level of trust in future health care. They trust certified alternative practitioners less than regular health care professionals, and non-certified alternative practitioners less still. The less trust patients have in future health care, the more they will be inclined to use CAM, when controlling for socio-demographic and disease characteristics.

**Conclusion:**

Trust in future health care is a significant predictor of CAM use. Chronically ill people's use of CAM may increase in the near future. Health policy makers should, therefore, be alert to the quality of practising alternative practitioners, for example by insisting on professional certification. Equally, good quality may increase people's trust in public health care.

## Background

Patients' trust in the health care system and trust in health care professionals are essential prerequisites for the effectiveness of health care [1-3]. Trust in health care is an indicator for support of the health care sector [4,5]. Trust in a health care professional is an important condition for a good relationship, therapeutic success and compliance [6]. In this article, trust was defined as trust in present health care, in future health care, in existing medical possibilities and in health care professionals.

Trust in health care has decreased in recent decades [6]. Higher education levels as well as abundant information both on new treatments and on physician and hospital errors are possible explanations for this decline in trust. However, in a more recent study it was concluded that trust in health care remains relatively stable [5]: Determinants of trust cited are a longer relationship with the physician, doctor's communication skills and doctor-patient interaction [7,8]. A lack of trust may cause patients to ask more often for a second opinion or to be more inclined to use complementary and alternative medicine (CAM) [7,9,10].

Trust in health care and health care professionals may be even more important for the chronically ill, because many of them must rely on health care and health care professionals in order to maintain their functional status, often for the rest of their life. Nearly all of them contact their general practitioner (GP) yearly, while three quarters of the general population visit their GP yearly [11,12]. Chronically ill people also contact a medical specialist more frequently than the general population: 76% and 40%, respectively [12].

Since chronic diseases are by definition (partly) incurable, it may be understandable that chronically ill people lose their trust in regular health care and turn to CAM, often alongside regular care. The popularity of CAM has grown significantly in all modern societies over the past two decades [13,14]. CAM is defined as all types of diagnostics and therapies not taught at medical faculties or official acknowledged paramedical training colleges [15]. About 33% of the chronically ill people use CAM, whereas only 6% of the general Dutch population and 12% of people with poor health (not only chronically ill people) use CAM [16-19].

In brief, patients report the following motives for using CAM [10,20-24]. They are dissatisfied with regular health care because doctors treat patients as a number instead of a person, and because they don't listen to the patient and they don't understand her or him. Another reason is the failure of conventional medicine; if doctors can no longer offer help towards recovery, people may turn to CAM, trying everything possible to improve their health, even as a last resort. In addition, people are looking for hope and they reason that 'if there is no benefit from CAM, there is no harm in trying CAM'. A further motive is that alternative practitioners typically have a holistic view, meaning that CAM emphasises the treatment of whole person, rather than just focusing on the symptom or the area that has the problem; according to the holistic approach, there are connections between body, mind and spirit, whereas mainstream medicine does not take this view. Moreover, people want a more active role in treatment and greater control. Lastly, a strong belief in CAM is cited as a motive. All these motives may be especially important for chronically ill people.

The most frequently cited chronic diseases for which patients are most likely to use CAM are musculoskeletal problems (especially low back pain), pain, headache/migraine, and rheumatoid arthritis. Within CAM, patients mainly use acupuncture, homeopathy, manual therapy (chiropractic), nutrition [14,21,25], and paranormal and naturopathic healers [15].

Many studies have shown that women, younger and highly educated patients, and those with a long illness duration, poor functional status and comorbidity use CAM more often than their counterparts [11-13,16,17,21,25-31]. Illness duration is related to CAM use because CAM is often seen as the last possible remedy [32].

Until now, no reports have been published about the relationships between trust in health care and health care professionals and CAM use by chronically ill people. In this study, we therefore examine the questions mentioned below.

### Research questions

1. To what extent do chronically ill people trust health care and health care professionals?

2. Is the extent of trust in health care and health care professionals related to socio-demographic and disease characteristics?

3. To what extent is the use of complementary and alternative medicine by chronically ill people predicted by their trust in health care and health care professionals, controlled for potentially confounding variables?

## Methods

### Sample

The present sample comprises respondents of the 'Panel of Patients with Chronic Diseases' (PPCD). PPCD is a nationwide research program investigating the consequences of chronic illness for patients and their families in the Netherlands [33]. Patients (N = 2484 at the onset) were recruited in 2001 via a representative sample of 51 general practices. The protection of the collected data was laid down in privacy regulations, safeguarding ethical consent, and registered by the Dutch Data Protection Authority (nr. 1283171).

The data presented were drawn from a postal questionnaire (October 2003), which was returned by 1,651 respondents (response 85%). For the purpose of this study, only patients of 25 years and older were included, since only a few respondents were aged between 15 and 24 years and it was assumed that patients of 25 years and older had already reached their highest educational level (education was included as determinant in the analyses). This yielded a total of 1,625 chronically ill people.

### Questionnaire

A questionnaire was used that had been developed earlier to assess – apart from socio-demographic and other basic characteristics – trust in health care and health care professionals and the use of complementary and alternative medicine (CAM) [2,34].

First, public trust in health care was measured by means of three items: trust in present health care, trust in future health care and trust in existing medical possibilities (the ability of medicine to treat problems effectively). The instrument 'public trust in the health care system' was developed to measure different dimensions of public trust in health care in the Netherlands. The instrument 'trust in present health care' was derived from six dimensions, comprising 36 items [34], indicating a general trust in health care (not only health care people have actually received or are receiving themselves). There are indications of the validity and reliability of this measurement instrument which have also been shown in later studies[2,4,5]. Trust in future health care (a general trust) and trust in existing medical possibilities, i.e., trust in the ability of medicine to treat problems effectively, were also found to be valid and reliable instruments in the studies just mentioned.

Patients indicated the extent of the three types of public trust on a 10-point scale (1 = no trust; 10 = complete trust). Secondly, interpersonal trust in five separate health care professionals was measured by means of a 4-point scale (1 = very little, 2 = little, 3 = much, 4 = very much); three regular health care professionals (general practitioners, medical specialists, physiotherapists), and two types of alternative practitioners: certified doctors practising CAM and alternative healers who practise CAM but who are not certified as a doctor.

In this article, CAM includes acupuncture, homeopathy, manual therapy (chiropractic), paranormal therapy, naturopathic therapy, anthroposophy and a remaining category.

Concerning CAM use, patients were asked to fill in whether they had ever used CAM or not. If they had used CAM, they were asked in what year and for which health problems they had used CAM. The period was dichotomised into past use (i.e., before 2002) and recent use (in 2002/2003).

Socio-demographic characteristics included gender, age, highest educational level finished (low = no/primary school, middle = secondary school, high = higher vocational training/university).

Disease characteristics concerned illness duration (in years); comorbidity (yes = more than one chronic disease); perceived functional status (functioning at home, at work and in leisure time as perceived by the chronically ill people;1 = excellent, 2 = very good, 3 = good, 4 = moderate, 5 = poor); and type of chronic disease.

For the purpose of the present study, eight diagnostic groups were distinguished on the basis of the patient's first diagnosis: musculoskeletal diseases, asthma/COPD, diabetes mellitus, cardiovascular diseases, neurological diseases, cancer, digestive diseases, and other chronic diseases.

### Statistical analyses

Analyses were carried out using the Statistical Package for Social Sciences (SPSS 11.5).

The data were weighted for the eight diagnostic groups; the reference group was the original panel of chronically ill people (2001, N = 2484).

Factor analyses were carried out to obtain scales measuring trust (scale 1). One factor could be distinguished consisting of trust in health care: trust in present and future health care and existing medical possibilities; explained variance 68.5%; factor loadings 0.88, 0.83 and 0.80, respectively; reliability α = 0.77. Factor analysis of trust in regular health care professionals and alternative practitioners showed two relevant scales explaining 65.2% of the total variance; (scale 2) consisting of trust in GPs, medical specialists, physiotherapists; factor loadings were 0.79, 0.81 and 0.67, respectively, reliability α = 0.63; (scale 3) trust in alternative practitioners: those who are certified as a doctor and those who are not certified as a doctor; factor loadings 0.88 and 0.87, respectively, reliability α = 0.70. (The factor loadings of the items of one scale were below 0.23 on the other scale.) Next, scale scores were calculated on the basis of these factors.

In order to answer the first and second research questions, descriptive statistics were computed: *t *tests for independent samples for pair wise comparisons, Chi-square and one-way analysis of variance (ANOVA).

For the third question, logistic regression analysis was performed to predict CAM use by trust in health care and health care professionals. In the logistic regression analysis, only data of patients who never used CAM versus those who had recently used CAM were included, since trust had also recently been measured. Socio-demographic and disease characteristics that were relevant according to the literature were controlled for.

In order to know whether multilevel logistic regression analysis was necessary, intra-class correlations were calculated. After all, the GPs whose patients were selected for the present sample, might influence the patients' attitude towards CAM.

## Results

### Sample characteristics

In total 1,625 chronically ill people of 25 years and older (mean age 60 years, SD 13.9) were included; 42.3% male and 57.7% female patients. Most of them had completed low or medium education (43.4% and 40.2%, respectively), 16.4% were highly educated (higher vocational training/university). About one third (34.2%) suffered from more than one chronic illness (comorbidity). About two thirds of the chronically ill people perceived their health status to be good to excellent, one third average and 6% poor. The average illness duration was 12 years (SD 9.8). Musculoskeletal diseases (17.8%), asthma/COPD (17.7%), diabetes mellitus (12.4%) and cardiovascular diseases (10.9%) were the chronic illnesses most often (first) diagnosed in the sample.

### Trust in health care and health care professionals

Patients' trust in present health care and in existing medical possibilities is high; 90% and 94%, respectively, give trust a satisfactory mark (i.e., 6 or higher), while 66% give a satisfactory mark to confidence in future health care.

Patients suffering from musculoskeletal diseases have (significantly) less confidence in present health care than those with cardiovascular diseases; they also trust future health care less than diabetes and cancer patients, and they have less trust in the existing medical possibilities than cancer patients (data not shown).

The great majority of chronically ill people do have (very) considerable trust in GPs and medical specialists, as well as in physiotherapists (Table 1), whereas a minority trust alternative, certified doctors. Only 12% of the chronically ill people trust alternative healers (who are not certified as a doctor), meaning that 88% do not trust them.

### Relationship between trust in health care and health care professionals and socio-demographic and disease characteristics

Patients' characteristics are partly related to their trust in health care and health care professionals and, if there are significant differences, these are sometimes rather small (Table 2). Male and older (> 65 years) chronically ill people and those perceiving their functional status as good to excellent have significantly more trust in health care than their counterparts.

Trust in regular health care professionals is higher when chronically ill people are older, less educated, well functioning, and suffering from more than one chronic illness.

The younger and higher educated the patients are, the higher their trust is in alternative practitioners. Furthermore, patients without comorbidity and a good functional status have more trust in CAM than those having more than one chronic illness and a poor functional status.

The type of chronic disease is not related to the trust chronically ill people have in health care and health care professionals, with two exceptions. Patients with musculoskeletal diseases have significantly less trust in health care overall than those suffering from cancer or diabetes (not shown in the table).

### The relationship between trust in health care and in regular health care professionals and alternative practitioners, and the use of complementary and alternative medicine

About one third of the chronically ill people (30%) reported ever having used CAM. Approximately half of them had used CAM in the past (more than two years ago, not recently) and the other half recently, i.e. 2003/2004 (52.4% and 47.6%, respectively).

Patients who have recently used CAM report less trust in present and future health care as well as in existing medical possibilities than those who have never used CAM (Table 3). The recent users have also less trust in future health care compared to patients who had used CAM in the past.

The chronically ill people who have never used CAM trust GPs more than the recent users, and the non-users trust medical specialists more than both past and recent users of CAM. However, these differences are rather small; recent CAM users also score high on trust in health care, GPs and medical specialists. Concerning physiotherapists, no differences in trust are found. Patients who have ever used CAM and especially the recent users have more trust in alternative practitioners than the non-users. Their trust is higher when the alternative practitioner is a doctor.

In order to predict CAM use by trust in health care and health care professionals, logistic regression analysis was performed (Table 4). The intra-class correlations of trust and CAM use did not significantly vary between GPs. Therefore, it was not necessary to perform multilevel multivariate logistic analyses.

Chronically ill people's trust in present health care and existing medical possibilities is not related to using CAM. However, the less patients trust health care in the future, the more they are inclined to use CAM: the chance becomes 0.75 higher if the score on trust in future health care is one point lower.

A higher level of trust in regular health care professionals decreases CAM use: the chance becomes 0.18 lower if the score on trust is one point higher. With respect to trust in alternative practitioners, the chance of using CAM is as much as almost eight times higher if the score on trust is one point higher.

Socio-demographic characteristics are also related to CAM use. The chance that female patients use CAM is more than three times higher than for male patients, and the younger the patients, the higher the chance they use CAM. Patients with a medium educational level are likely to use CAM about twice as often as chronically ill people with a low education.

Illness duration, comorbidity and functional status are not associated with CAM use when controlling for other characteristics. Compared to the group of patients with 'other' chronic diseases, patients suffering from cardiovascular diseases, diabetes and asthma/COPD are less likely to use CAM.

## Discussion

The use of complementary and alternative medicine by chronically ill people is best explained by the fact that the chronically ill people are somewhat pessimistic about the future of Dutch health care. Chronically ill people's confidence in present health care and in existing medical possibilities is not related to using CAM. Chronically ill people have a relatively low level of trust in future health care. They trust certified alternative practitioners less than regular health care professionals, and non-certified alternative practitioners less still.

The relatively low confidence in future health care might be related to current social developments, such as the change of the (Dutch) financing system of health care and the higher costs arising from this change; the change of institutions that are responsible for payments; and the cuts in reimbursements of costs. Moreover, non-financial matters may play a role, such as waiting lists and the quality of care delivered by institutions.

It seems contradictory, that (chronically ill) people have more trust in the existing medical treatment possibilities than in future health care, because these possibilities are still increasing. Gene therapy, for example, seems a promising treatment for some chronic diseases, such as diabetes and certain digestive illnesses. Likewise, progress is being made in the area of neurological diseases and cancer research. Maybe, chronically ill people attach more influence to social developments as cited above than to the the growth of medical treatment possibilities, when they are asked for their level of trust in future health care.

Maybe, the progress is not fast enough from their perspective, the more because most chronically ill people are seniors and they do not expect to receive the benefits of new treatments during their own life.

Trust in present health care and in trust in existing medical treatment possibilities is about equal. However, the concepts are different; trust in present health care is a broad concept and trust in medical possibilities is specifically aimed at treatments. Trust in present health care includes both cure and care. Care is often as much as important as cure, especially for chronically ill people who can not recover from their illness and who, therefore, try CAM. Empathy of health professionals and other affective talk, as well as a good communication and a good relationship with caregivers are very important for the quality of life [35-37]. Therefore, health care professionals should be educated in communication skills.

People may consult alternative practitioners because of their better understanding and holistic view, which makes them feel more comfortable and better understood. Regular health care professionals might learn from their alternative colleagues in these respects, e.g., by additional education, in order to improve public health.

Regular health care professionals are trusted well by nearly all chronically ill people, but only a minority of them trust alternative doctors, and only one out of ten trust alternative healers (not doctors). Compared to a recent study among the general population [38], chronically ill people have about the same level of confidence in health care professionals, both regular and alternative, as people without a chronic disease. Apparently, trust in health care professionals is a generalized attitude among all people, irrespective of diseases.

This study supports earlier studies with regard to the relationship between socio-demographic characteristics (gender, age and educational level) and the level of trust, which is in line with earlier findings among the general population [38]. However, because the differences are sometimes minor, their practical relevance should not be overestimated.

The use of complementary and alternative medicine is relatively high among chronically ill people. One third of the chronically ill people in the present study have ever used CAM, which corresponds with an earlier study [17]. In comparison, 6% of the general population and 12% of people with a poor physical health use CAM [16,17,39].

Patients with a chronic disease who have a high level of trust in regular health care professionals will be less inclined to use CAM, whereas those who trust alternative practitioners will choose CAM much more often. This is of course logical. Interestingly, disease characteristics like illness duration, comorbidity and functional status are not related to CAM use if trust is controlled for. This is contrary to earlier findings [11-13,16,17,21,25-31]. Maybe, this is due to the inclusion in the analysis of chronically ill people who have used CAM in 2002 and 2003, instead of chronically ill people who have ever used CAM. Another reason for this difference may be the composition of the present sample, i.e. chronically ill people in general, instead of specific groups of chronically ill people.

Some chronic diseases are more likely to be presented to alternative practitioners than others. This may be due to the relative effectiveness of the alternative treatment for the disease in question. People with digestive and musculoskeletal diseases, for example, may find benefit in treatments such as alternative diets and manual therapy, respectively [40-44], while other chronic diseases are virtually incurable. However, contradictory results of CAM use for chronic diseases have also been found [45-48]. More research into the CAM use for specific chronic diseases might be helpful.

Younger and well-educated chronically ill people may be more inclined to use CAM than older and less-educated ones, which was also found in earlier studies. CAM use might increase even more, since more people will be highly educated in future. Apart from the relatively low trust in future health care as mentioned above, other reasons for higher future CAM use could be that many chronically ill people see CAM as complementary to regular health care, and that CAM is increasingly being offered by practitioners who are certified [20,22,49]. The stronger trust in alternative practitioners who are also doctors seems logical, because these doctors are capable of integrating regular and alternative medical knowledge and principles in their treatments. Health care policy should, therefore, aim at certifying alternative practitioners, so that people can really trust them.

Whether a higher CAM use is a favourable development depends on the way CAM is used and applied. Health policy makers should be on the alert for malpractice by alternative practitioners and should strive for a quality assessment of practising alternative practitioners, just as is the case for regular health care professionals. As a consequence, a good quality of health care may increase people's trust in both regular and complementary, alternative health care.

## Conclusion

The conclusion is that trust is indeed an important concept in the use of health care by chronically ill people. The present study reveals that trust in future health care is a significant predictor of the use of complementary and alternative medicine. If the relatively low trust in future health care should continue, the use of complementary and alternative medicine may increase in the near future, at least by chronically ill people. Health policy makers should, therefore, be alert to the quality of practising alternative practitioners, for example by insisting on professional certification. In turn, a good quality may increase people's confidence in public health care.

## Competing interests

The author(s) declare that they have no competing interests.

## Authors' contributions

AB-M participated in the design of the study, performed the statistical analysis and drafted the manuscript. PMR conceived of the study, participated in its design and helped to draft the manuscript. Both authors read and approved the final manuscript.

## Pre-publication history

The pre-publication history for this paper can be accessed here:



## Figures and Tables

**Table 1:** Chronically ill people's trust (%) in health care professionals (N = 1625)

Trust in:	Score < 6	Score ≥ 6
*Regular health care professionals:*		
- GPs	14.3	85.7
- medical specialists	11.3	88.7
- physiotherapists	17.5	82.5
*Alternative practitioners:*		
- alternative doctors	56.9	43.1
- alternative healers (not doctors)	88.3	11.7

**Table 2:** Chronically ill people's trust (mean, standard deviation) in health care and health care professionals by socio-demographic and disease characteristics (N = 1625)

	Trust in health care (range 1–10)	Trust in regular health care professionals (range 1–4)	Trust in alternative practitioners (range 1–4)
*Total*	6.70 (1.05)	2.99 (0.44)	2.00 (0.65)
*Gender:*			
- men	6.85 (1.05)*	3.00 (0.43)	1.99 (0.66)
- women	6.59 (1.04)	2.98 (0.45)	2.00 (0.64)
*Age:*			
group 1: < 50 years	6.65 (1.06)^3^	2.95 (0.39)^3^	2.11 (0.64)^2,3^
group 2: 50–65 years	6.56 (1.06)^3^	2.96 (0.46)^3^	2.00 (0.61)^1,3^
group 3: > 65 years	6.90 (1.00)^1,2^	3.04 (0.44)^1,2^	1.90 (0.68)^1,2^
*Educational level:*			
group 1: low	6.72 (1.08)	3.03 (0.46)^2,3^	1.89 (0.66)^2,3^
group 2: medium	6.66 (1.02)	2.96 (0.43)^1^	2.04 (0.63)^1^
group 3: high	6.74 (1.07)	2.95 (0.42)^1^	2.13 (0.62)^1^
*Illness duration:*			
group 1: < 5 years	6.74 (1.00)	2.94 (0.45)	1.97 (0.63)
group 2: 5–9 years	6.70 (1.09)	2.97 (0.44)	1.99 (0.66)
group 3: ≥10 years	6.69 (1.06)	3.01 (0.45)	2.00 (0.65)
*Comorbidity:*			
- no	6.71 (1.03)	2.96 (0.43)*	2.03 (0.65)*
- yes	6.70 (1.10)	3.03 (0.46)	1.93 (0.64)
*Functional status:*			
- good to excellent	6.82 (0.99)*	3.01 (0.41)*	2.05 (0.63)*
- moderate to poor	6.52 (1.14)	2.95 (0.49)	1.91 (0.66)
*Type of chronic disease:*			
musculoskeletal diseases	6.48 (1.21)	2.93 (0.47)	1.96 (0.61)
asthma/COPD	6.70 (1.04)	2.99 (0.42)	2.01 (0.63)
Diabetes	6.88 (1.13)	3.02 (0.44)	2.02 (0.71)
cardiovascular diseases	6.76 (0.86)	3.01 (0.43)	1.93 (0.61)
neurological diseases	6.66 (0.94)	2.99 (0.46)	2.00 (0.66)
Cancer	7.03 (0.88)	3.06 (0.41)	1.93 (0.76)
digestive diseases	6.70 (0.68)	2.91 (0.34)	1.91 (0.61)
other chronic diseases	6.70 (1.04)	2.99 (0.44)	2.04 (0.62)

* P ≤ 0.05 compared to the reference group

^1 ^score differs significantly (P ≤ 0.05) from the score of group 1

^2 ^score differs significantly (P ≤ 0.05) from the score of group 2

^3 ^score differs significantly (P ≤ 0.05) from the score of group 3

**Table 3:** Chronically ill people's trust in health care and health care professionals (mean, standard deviation) by CAM use

Trust in:	1. Never used CAM (N = 1093)	2. Used CAM in the past (N = 245)	3. Used CAM recently (N = 223)
*Health care*^a^:	6.8 (1.0)^3^	6.6 (1.1)	6.4 (1.0)^1^
- present health care	7.0 (1.2)^3^	6.9 (1.2)	6.7 (1.1)^1^
- future health care	6.0 (1.5)^3^	5.9 (1.5)^3^	5.6 (1.4)^1,2^
- existing medical possibilities	7.3 (1.1)^3^	7.1 (1.2)	7.0 (1.1)^1^
*Regular health care professionals*^b^:	3.0 (0.4)^3^	3.0 (0.5)	2.9 (0.4)^1^
- GPs	3.1 (0.6)^3^	3.0 (0.6)	2.9 (0.6)^1^
- medical specialists	3.1 (0.5)^2,3^	3.0 (0.6)^1^	2.8 (0.6)^1^
- physiotherapists	2.9 (0.6)	2.9 (0.5)	2.8 (0.6)
*Alternative practitioners*^b^:	1.8 (0.6)^2,3^	2.1 (0.6)^1,3^	2.4 (0.6)^1,2^
- alternative doctors	2.2 (0.7)^2,3^	2.5 (0.7)^1,3^	2.8 (0.7)^1,2^
- alternative healers (not doctors)	1.5 (0.7)^2,3^	1.8 (0.7)^1,3^	2.1 (0.8)^1,2^

^a ^score 1–10

^b ^score 1–4

^1 ^score differs significantly (P ≤ 0.05) from the score of patients who never used CAM

^2 ^score differs significantly (P ≤ 0.05) from the score of patients who used CAM before 2002

^3 ^score differs significantly (P ≤ 0.05) from the score of patients who used CAM in 2002/2003

**Table 4:** Odds ratios and confidence intervals (95% CI) of the chance that chronically ill people will use CAM (N = 944)

*Trust in:*	OR	95% CI
- present health care (score 1–10)	1.15	0.91 – 1.46
- future health care (score 1–10)	0.73	0.61 – 0.87
- medical possibilities (score 1–10)	1.16	0.93 – 1.46
- regular health care professionals (score 1–4)	0.18	0.10 – 0.33
- alternative practitioners (score 1–4)	8.23	5.63 – 12.04
*Socio-demographic characteristics:*		
- gender (1 = female)	3.35	2.13 – 5.26
- age (years)	0.98	0.96 – 0.99
- medium educational level^1^	2.12	1.32 – 3.40
- high educational level^1^	1.78	0.98 – 3.23
*Disease characteristics:*		
- illness duration (years)	1.01	0.98 – 1.02
- comorbidity (1 = yes)	1.38	0.88 – 2.18
- functional status (1 = excellent, 5 = poor)	1.19	0.93 – 1.53
Type of chronic disease:^2^		
- musculoskeletal diseases	0.95	0.54 – 1.67
- asthma/COPD	0.39	0.20 – 0.76
- diabetes	0.48	0.24 – 0.98
- cardiovascular diseases	0.22	0.08 – 0.58
- neurological diseases	0.52	0.23 – 1.15
- cancer (not in a terminal stage)	0.93	0.37 – 2.32
- digestive diseases	1.31	0.44 – 3.91

^1 ^reference group: low educational level

^2 ^reference group: other chronic diseases
